# Two new species of *Quedius* Stephens, subgenus *Raphirus* Stephens from Yunnan, Southwest China (Coleoptera, Staphylinidae, Staphylinini)

**DOI:** 10.3897/zookeys.165.2331

**Published:** 2012-01-13

**Authors:** Jia-Yao Hu, Li-Zhen Li, Guang-Hong Cao

**Affiliations:** 1 Department of Biology, Shanghai Normal University, Shanghai, 100 Guilin Road, Shanghai, 200234 P. R. China; 2 Administration of Nabanhe River Watershed National Nature Reserve, Jinghong 666100, P. R. China

**Keywords:** Coleoptera, Staphylinidae, Staphylinini, *Quedius*, key, Yunnan, Southwest China, new species

## Abstract

Two new species of the genus *Quedius* Stephens collected from Nabanhe Nature Reserve, Yunnan Province, *Quedius nabanhensis* **sp. n.** and *Quedius maoxingi* **sp. n.**, are described and illustrated. Keys to the *multipunctatus* group and *intricatus* group of *Quedius* species of Yunnan are provided. A map of the collecting sites is given.

## Introduction

To date, about 50 species of the genus *Quedius* Stephens, 1829 have been known from Yunnan Province, China (Herman 2001 and subsequent papers: Smetana 2002, 2004, 2008a, 2008b, 2008c, 2009, 2011). Among them, four species belong to the *multipunctatus* species group (*Quedius chrysogonus* Smetana, 1997a, *Quedius puetzi* Smetana, 1998, *Quedius viridimicans* Smetana, 2009 and *Quedius michaeli* Smetana, 2009) and two to the *intricatus* species group (*Quedius rivulorum* Smetana, 2002 and *Quedius torrentum* Smetana, 2002), both species groups from the subgenus *Raphirus* (Smetana 1995). The recent expedition to Nabanhe Nature Reserve, south Yunnan Province, brought two new species of the genus, which are described below: *Quedius nabanhensis* sp. n. and *Quedius maoxingi* sp. n. Both of them are placed in the subgenus *Raphirus* Stephens, 1829: the former to the *multipunctatus* group and the latter to the *intricatus* group. Both new species are here described and illustrated. Keys to the species of *Quedius* from Yunnan belonging to the *Multipunctatus* and *Intricatus* species groups species are provided. A map (Fig. 16) of the collecting sites is given. Holotypes and most of the paratypes are deposited in the Insect Collection of Shanghai Normal University, Shanghai, P. R. China. Two paratypes of *Quedius nabanhensis* is deposited in the collection of Dr. Aleš Smetana (Ottawa, Canada).

## Methods

The specimens were collected by sifting from wet moss on rocks in streams. They were killed with ethyl acetate and dried. The sternites, tergites and aedeagi were mounted in the Euparal on plastic slides. The habitus photos were taken with a Canon 40D camera. The photos of the sternites, tergites and aedeagi were taken with a Canon G9 camera mounted on an Olympus SZ61 stereoscope.

### Measurements

Body length: measured from the anterior margin of the labrum to the end of abdomen;

Forebody length: measured from the anterior margin of the labrum to the elytral apices;

Head length: measured from the apical margin of the head capsule to its posterior margin;

Head width: Maximal width of the head across eyes;

Eye length: longitudinal length of the eye in dorsal view;

Tempora length: length of the tempora in dorsal view;

Pronotum length: measured from the front margin of the pronotum to its posterior margin along the imaginary median line;

Pronotum width: width of the pronotum across its widest part;

Elytral width: width of the elytra at base;

Elytral length at suture: measured from the apex of the scutellum to the apex of suture;

Elytral length at lateral margins: measured from the humeral angle to the elytral apex.

## Descriptions

### Quedius nabanhensis sp. n.

urn:lsid:zoobank.org:act:39047DBA-1AD7-44D0-9C0F-CBB27EB8A71B

http://species-id.net/wiki/Quedius_nabanhensis

Figs 1–8

#### Type material.

Holotype. CHINA: Yunnan Prov.: male, Jinghong City, Nabanhe Nature Reserve, Bengganghani, Huazhulianshan, alt. 2,300 m, 29-IV-2009, Jia-Yao HU & Zi-Wei YIN leg. Paratypes. 3 males, 3 females, same data as holotype.

#### Description.

Body length: 8.0–8.6 mm; forebody length: 4.1–4.3 mm.

**Figures 1–8.**
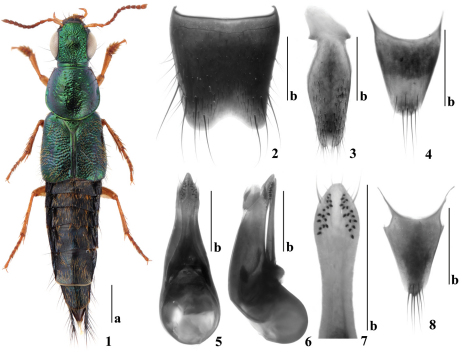
*Quedius nabanhensis* sp. n. **1** habitus **2** male sternite VIII **3** male sternite IX **4** male tergite X **5** aedeagus in ventral view **6** aedeagus in lateral view **7** apical portion of underside of paramere **8** female tergite X. Scale bars: a = 1mm, b = 0.5mm.

Body (Fig. 1) shiny, head, pronotum and elytra bright metallic green, abdomen black and iridescent; appendages yellowish brown, antennae slightly darkened from base toward apex.

Head slightly wider than long (ratio 1.18); eyes large and convex, tempora distinctly shorter than eyes from above (ratio 0.18); dorsal surface of head with coarse and dense punctation, becoming finer toward vertex and clypeus; surface with fine and dense microsculpture of transverse waves, becoming almost meshed anterior to vertex. Antennae slightly widened apicad, segments III slightly longer than II, segments IV–IX longer than wide, gradually becoming shorter, segments X about as long as wide, last segment as long as two preceding segments combined.

Pronotum about as long as wide; dorsal rows each with seven to eight coarse punctures, forming irregular row; sublateral rows each expanded into irregular group of seven to ten punctures; some distinctly finer punctures scattered among coarse punctation; surface with fine and dense microsculpture of transverse waves. Scutellum with nine to fourteen fine punctures, with fine microsculpture of transverse waves. Elytra at base about as wide as pronotum at widest point, at suture slightly shorter (ratio 0.84), at lateral margins slightly longer (ratio 1.35) than pronotum at midline; punctation coarse and dense, almost confluent and forming transverse rugae; pubescence yellowish-golden; surface without microsculpture. Wings fully developed.

Abdomen with tergite VII bearing distinct whitish apical seam of palisade fringe; punctation moderately fine and dense; pubescence black, each tergite with some scattered golden pubescence; surface with fine and dense microsculpture of transverse waves.

*Male*. First four segments of protarsus distinctly dilated. Sternite VIII (Fig. 2) with four long setae on each side, with wide, shallow, arcuate medioapical emargination. Sternite IX (Fig. 3) slightly notched in medioapical emargination, with two differentiated apical setae. Tergite X (Fig. 4) with several apical setae. Aedeagus (Figs 5–7) with median lobe gradually narrowed into cone-shaped apex; paramere not reaching apex of median lobe, in ventral view distinctly narrowed at middle and slightly widened near apex; with two apical setae, two similar setae and one long seta at each lateral margin below apex; underside of paramere with sensory peg setae arranged into two longitudinal areas, each with 16–17 peg setae.

*Female*. First four segments of protarsus similar to those of male, but distinctly less dilated. Tergite X (Fig. 8) with several apical setae.

#### Distribution.

Known only from the type locality (Southwest China: Yunnan Province).

#### Remarks.

*Quedius nabanhensis* is closest to *Quedius xeno* Smetana, 1997b, the species known from Northern Vietnam based on the female holotype only, due to the similar form and color of the body in both species. The new species can be distinguished from *Quedius xeno* by the larger size (8.0–8.6 mm), the scutellum with several punctures and by the female tergite X (Fig. 8) with several apical setae. *Quedius xeno* is smaller (6.6 mm), the scutellum lacks punctures and the female tergite X bears with two setae.

#### Etymology.

The specific epithet is derived from the type locality.

### Quedius maoxingi sp. n.

urn:lsid:zoobank.org:act:8EB20BBE-E5E1-468F-9F27-FA5BAA78B815

http://species-id.net/wiki/Quedius_maoxingi

Figs 9–15

#### Type material.

Holotype. CHINA: Yunnan Prov.: male, Jinghong City, Nabanhe Nature Reserve, Guomenshan, alt. 1,200 m, 8-V-2009, Jia-Yao HU & Zi-Wei YIN leg.

#### Description.

Body length: 6.8 mm; forebody length: 3.4 mm.

**Figures 9–15.**
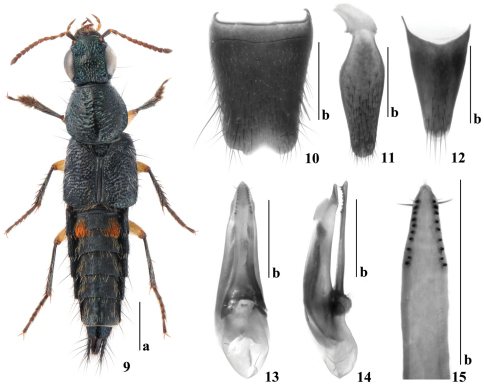
*Quedius maoxingi* sp. n. **9** habitus **10** male sternite VIII **11** male sternite IX **12** male tergite X **13** aedeagus in ventral view **14** aedeagus in lateral view **15** apical portion of underside of paramere. Scale bars: a = 1mm, b = 0.5mm.

Body (Fig. 9) shiny, head, pronotum and elytra dark metallic blue, abdomen black and iridescent; maxillary and labial palpi brown with basal segments slightly paler; antennae brown, first segments and apical halves of following two segments black; legs black with femora and front coxae yellowish.

Head slightly wider than long (ratio 1.12); eyes large and convex, tempora distinctly shorter than eyes seen from above (ratio 0.18); dorsal surface of head with coarse and dense punctation; clypeus and vertex lacking punctures; surface with fine and dense microsculpture of transverse waves, becoming almost meshed anterior to vertex. Antennae slightly widened toward apex, segments II and III subequal in length, IV and V slightly longer than wide, segments VI–X about as long as wide, last segments about as long as two preceding segments combined.

Pronotum about as long as wide; dorsal rows irregular, each with 16 coarse punctures, each row expanding into group of punctures posteriorly; sublateral rows each expanded into irregular group of nine to ten coarse punctures; some distinctly finer punctures scattered among coarse punctation; with many dense and fine punctures bearing whitish hairs in wide strip along lateral margin; surface with fine and dense microsculpture of transverse waves. Scutellum with 14 punctures, with fine microsculpture of transverse waves. Elytra at base about as wide as pronotum at widest point, at suture slightly shorter (ratio 0.74), at sides slightly longer (ratio 1.26) than pronotal midline; punctation coarse and dense, on disc forming transverse rugae; pubescence dark, intermixed with whitish hairs, particularly on lateral portion of each elytron; surface without microsculpture. Wings fully developed.

Abdomen with tergite VII bearing distinct whitish apical seam of palisade fringe; punctation moderately fine and dense; tergite III with distinct tuft of golden-reddish tomentose pubescence on each lateral portion; pubescence black at middle portion, with some whitish hairs at both lateral portions and at apical margin of each tergite; surface with fine and dense microsculpture of transverse waves.

*Male*. First four segments of front tarsus distinctly dilated. Sternite VIII (Fig. 10) with two long setae on each side, with wide, shallow, arcuate medioapical emargination. Sternite IX (Fig. 11) simply rounded in medioapical emargination, without differentiated setae. Tergite X (Fig. 12) with five long setae near posterior margin and several shorter setae anterior to them; Aedeagus (Figs 13–15) with median lobe gradually narrowed into cone-shaped apex; paramere extending slightly beyond apex of median lobe; with two setae at apex, two slightly shorter setae and one distinctly longer seta at each lateral margin below apex; underside of paramere with sensory peg setae forming two regular longitudinal rows, each with nine or ten peg setae.

*Female*. Unknown.

#### Distribution.

Known only from the type locality (Southwest China: Yunnan Province).

#### Remarks.

*Quedius maoxingi* is closest to *Quedius barbarossa* Smetana, 2002 due to similar form and color of the body. The new species can be distinguished from *Quedius barbarossa* by the pronotum with some finer punctures scattered among coarse punctation, the scutellum with several punctures and by the aedeagus with symmetrical paramere (Fig. 15). *Quedius barbarossa* lacks fine punctures scattered among coarse punctation, its scutellum lacks punctures and the aedeagus bears distinctly asymmetrical paramere.

#### Etymology.

The species is named in honor of Maoxing Tian (Administration of Nabanhe River Watershed National Nature Reserve) who helped a lot during our collection in Yunnan.

##### Key to the *multipunctatus* species group of *Quedius* (*Raphirus*) from Yunnan Province, China

**Table:** 

1	Scutellum with several punctures; pubescence of abdominal tergites not uniform, bearing some golden hairs intermixed with black ones	*Quedius nabanhensis* sp. n.
–	Scutellum impunctate; pubescence of abdominal tergites uniform, black or yellowish golden	2
2	Pubescence of abdominal tergites black	3
–	Pubescence of abdominal tergites yellowish golden	4
3	Head and pronotum with dark green metallic lustre, elytra brilliant dark green; head with microsculpture gradually changing from transverse to meshed on anterior half	*Quedius chrysogonus* Smetana
–	Head and pronotum with bronze-green metallic lustre, elytra brilliant bronze-green; head with microsculpture barely changing from transverse to meshed on anterior half	*Quedius puetzi* Smetana
4	Pronotum with sublateral rows each expanded into irregular group of seven or eight punctures; sensory peg setae of underside of paramere forming two separate longitudinal rows	*Quedius viridimicans* Smetana
–	Pronotum with sublateral rows each expanded into irregular group of ten to twelve punctures;sensory peg setae of underside of paramere forming apical field below apex of paramere, then extended posteriad as a row along each lateral margin	*Quedius michaeli* Smetana

##### Key to the *intricatus* species group of *Quedius* (*Raphirus*) from Yunnan Province, China

**Table:** 

1	Femora entirely yellowish; scutellum with several punctures	*Quedius maoxingi* sp. n.
–	Apices and dorsal edge of femora black; scutellum impunctate	2
2	Abdominal tergite III with a tuft of golden-reddish tomentose pubescence on each lateral portion;sensory peg setae of underside of paramere forming a dense subapical field, with about 50 peg setae	*Quedius torrentum* Smetana
–	Abdominal tergite III without tufts of golden-reddish tomentose pubescence;sensory peg setae of underside of paramere forming two irregular longitudinal rows, each with 11 and 13 peg setae	*Quedius rivulorum* Smetana

**Figure 16.**
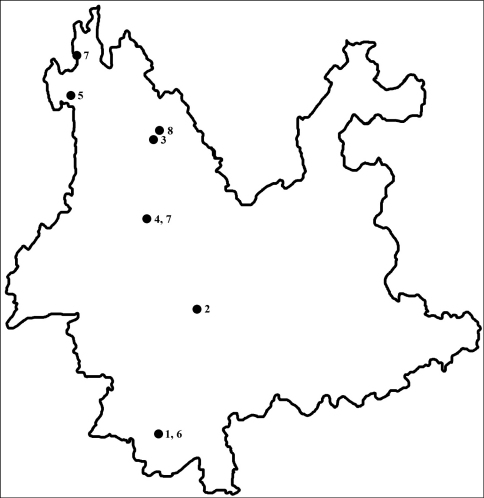
Map showing the collecting sites of the *multipunctatus* group and *intricatus* group of *Quedius* in Yunnan Prov. **1** *Quedius nabanhensis* sp. n. **2** *Quedius chrysogonus* Smetana **3** *Quedius puetzi* Smetana **4** *Quedius viridimicans* Smetana **5** *Quedius michaeli* Smetana **6** *Quedius maoxingi* sp. n. **7** *Quedius torrentum* Smetana **8** *Quedius rivulorum* Smetana.

## Supplementary Material

XML Treatment for Quedius nabanhensis

XML Treatment for Quedius maoxingi
